# The effect of different amounts of vitamin D supplementation on serum calcidiol, anthropometric status, and body composition in overweight or obese nursing women: a study protocol for a randomized placebo-controlled clinical trial

**DOI:** 10.1186/s13063-019-3622-y

**Published:** 2019-08-30

**Authors:** Zohre Gerveieeha, Fereydoun Siassi, Mostafa Qorbani, Farzaneh Ziaeian, Gity Sotoudeh

**Affiliations:** 1 Department of Community Nutrition, School of Nutritional Sciences and Dietetics, Tehran University of Medical Sciences, Hojatdost street, Naderi street, Keshavarz Blvd, Tehran, Iran; 2 Non-communicable Diseases Research Center, Alborz University of Medical Sciences, Karaj, Iran; 3 Chronic Diseases Research Center, Endocrinology and Metabolism Population Sciences Institute, Tehran University of Medical Sciences, Tehran, Iran; 4 Department of Health Science in Nutrition, Islamic Azad University Science and Research Branch of Tehran, Tehran, Iran

**Keywords:** Nursing women, Vitamin D, Overweight, Obesity

## Abstract

**Background:**

The optimal vitamin D intake for nursing mothers with overweight or obesity has not been defined. Vitamin D concentrations are associated with body composition indices, particularly body fat mass. Few studies have investigated the relationship between hypovitaminosis D, obesity, anthropometric status, and body composition in nursing women. Thus, the present study aims to investigate whether vitamin D supplementation during lactation will improve vitamin D status, reduce body fat mass, and improve body composition.

**Methods/design:**

In a double-blind, randomized, placebo-controlled, parallel-group trial, after term delivery, 90 healthy women with overweight or obesity will be selected and randomly allocated into three groups to receive 2000 IU/d cholecalciferol (vitamin D3), 4000 IU/d cholecalciferol, or placebo (lactose) for 12 weeks while nursing. Measurements of height, weight, waist circumference, and body composition (fat mass (kg), lean mass (kg), body fat (%), fat mass index, and relative fat mass index) will be taken for all subjects at baseline and after 12 weeks of intervention. In addition, serum 25-hydroxyvitamin D (25(OH)D), parathyroid hormone, calcium, and phosphorus will be measured.

**Discussion:**

This study is the first investigating the effect of different amounts of vitamin D supplementation on serum calcidiol, anthropometric status, and body composition in nursing women with overweight or obesity. Our findings will contribute to the growing body of knowledge regarding the role of vitamin D supplementation in obesity, anthropometric status, and body composition in nursing women.

**Trial registration:**

Iranian Registry of Clinical Trials IRCT20140413017254N6. Registered on 11 April 2018.

**Electronic supplementary material:**

The online version of this article (10.1186/s13063-019-3622-y) contains supplementary material, which is available to authorized users.

## Background

In developed countries, 50% of nursing mothers suffer from overweight or obesity [1]. In Iran, the prevalence of overweight or obesity in nursing women is estimated at 31.7–37.3% [2, 3]. Nursing mothers expend 500 kcal per day for milk production [4], resulting in gradual weight loss [5, 6].

However, factors such as unhealthy dietary intake, low physical activity [7], higher leptin level [8], or pre-pregnancy body mass index (BMI) [9], and deficiency of serum 25-hydroxyvitamin D (25(OH)D; ≤ 20 ng/ml or 50 nmol/l) [10] are associated with more weight gain or obesity during pregnancy, which may lead to chronic diseases in nursing mothers [11].

The global prevalence of vitamin D deficiency (≤ 20 ng/ml) and severe deficiency (≤ 10 ng/ml or 25 nmol/l) in pregnant and nursing women is estimated to be between 21 and 85% [12, 13]. The results of the Second National Survey on the status of micronutrients in Iran showed that 85% of pregnant women are exposed to vitamin D deficiency (≤ 20 ng/ml) or severe deficiency (≤ 10 ng/ml) [14]. Therefore, vitamin D deficiency is also expected to be high in nursing women. On the other hand, some studies have shown a decrease in serum levels of vitamin D during lactation [12, 15]. The retention of vitamin D in body fats due to overweight in pregnancy [16], an increase in the body’s need for bone mass [17], lack of adequate sunlight exposure [18], and very low levels of vitamin D intake are the most significant reasons for deficiency of vitamin D in nursing mothers [19].

Obesity and overweight are among the most important risk factors for vitamin D deficiency [20]. The concentration of 25(OH)D is low in adults with obesity [21] and is inversely related to body weight, BMI, percentage of body fat, and visceral fat [22, 23]. It has been reported that after exposure to sunlight, the increase in serum levels of vitamin D was 57% less in subjects with obesity compared to subjects without obesity. Thus, it has been concluded that the release of vitamin D from the skin into the blood is altered in individuals with obesity [12]. However, another study reported that volumetric dilution of vitamin D in the large adipose tissue causes vitamin D deficiency in individuals with obesity [24].

The Institute of Medicine (IOM) recommended 600 IU vitamin D for nursing women, which is based on the role of vitamin D in bone health, but treating women with obesity may require more amounts of vitamin D. The Endocrine Society guidelines recommend that adults with obesity need two to three times more vitamin D (6000–10,000 IU/d) to treat and prevent vitamin D deficiency [19].

The results of a systematic review reported that findings on the effect of vitamin D supplementation on weight reduction in subjects with overweight and obesity were not conclusive [25]. However, most of the included studies in this review used a combined treatment of vitamin D and other substitutes, making interpretation of the findings more difficult. Another systematic review and meta-analysis reported that 25(OH)D levels are inversely correlated with body fat percentage (BFP), but no effect was found regarding vitamin D supplementation on BFP [26]. However, many included studies in this meta-analysis were not performed specifically in individuals with overweight or obesity and included postmenopausal women or patients with type 2 diabetes mellitus. In addition, some of these studies did not use high doses of vitamin D. On the other hand, a review study concluded that most clinical trials with null effect of vitamin D on health were performed in populations without vitamin D deficiency. So possible beneficial effects from vitamin D supplementation cannot be excluded [27].

To the best of our knowledge, the effect of different doses of vitamin D supplementation on anthropometric status and body composition has not been investigated specifically in nursing women with overweight or obesity. Only one study investigated the effect of vitamin D supplementation on body composition in nursing women with different weight statuses, including women with overweight or obesity. However, data for women with obesity has not been presented separately. Receiving 400 or 1200 IU/d of vitamin D for 6 months has not been found to influence body composition, which is probably due to the low amount of the vitamin. However, an inverse relationship has been reported between serum level of vitamin D and fat mass [28]. Therefore, further studies are needed to determine the appropriate amount of vitamin D for improving vitamin D status and body composition in nursing mothers with overweight or obesity. Thus, we proposed to start a randomized controlled trial with the following objectives:

i. To investigate whether vitamin D supplementation can improve anthropometric status and body composition in nursing women with overweight or obesity
ii. To investigate whether vitamin D supplements can improve the vitamin D status of these women
iii. To investigate what amount of vitamin D supplementation can reduce the serum level of parathyroid hormone (PTH) in these women

## Methods/design

### Study design

**Fig. 1:**
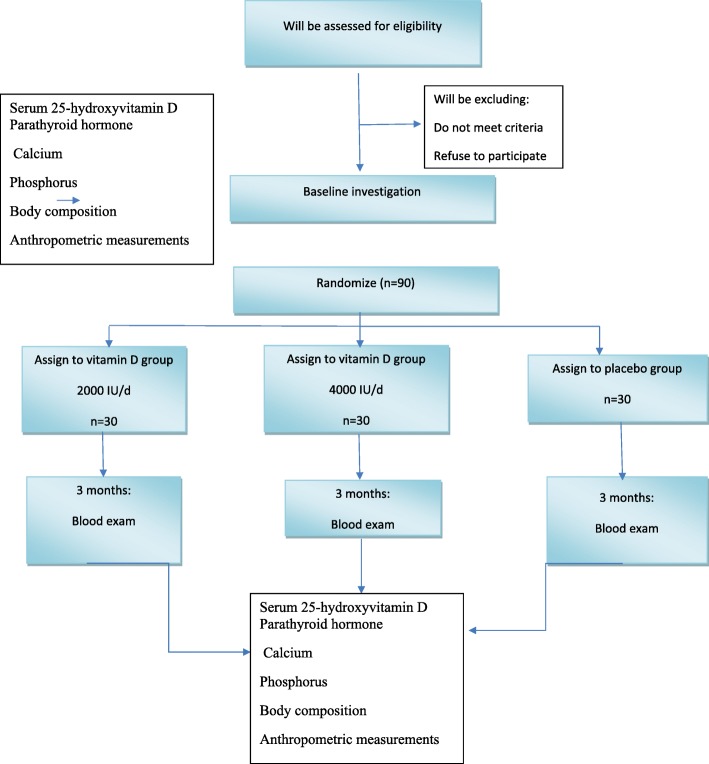
Recruitment flow chart

Subjects will be randomly assigned into groups with a 1:1:1 randomization ratio. A flow chart of the study protocol is presented in Fig. 1.

Four types of questionnaires will be completed by each subject at the beginning and end of the study: socio demographic, 24-h recalls to collect dietary intake data, sunlight exposure questionnaire, and physical activity questionnaire (International Physical Activity Questionnaires (IPAQ)).

The necessary information on anthropometric measurements (height, weight, BMI, body fat percentage, body weight (kg), lean mass (kg), visceral fat, and visceral fat mass (kg), relative fat mass index (RFM), fat mass index (FMI) will be obtained using In Body model 270 as a body composition analyzer (In Body Co., Ltd, Seoul, Korea). Serum levels of vitamin D, PTH, as well as calcium and phosphorus levels will be measured at the beginning and end of intervention. Table 1 shows the primary and secondary outcome measures and the time for measurement and a Standard Protocol Items: Recommendations for Interventional Trials (SPIRIT) checklist is included as Additional file 1. The anthropometric and body composition measures, including height, weight, BMI, body fat percentage, body weight, lean mass, visceral fat and visceral fat mass, RFM, and FMI were intended to be primary outcomes but were unintentionally omitted in the preregistration. However, our sample size calculation is based on waist circumference as intended as a primary measure.

**Table 1:** Outcome measures and measurement times

	Months	
	0	3
Primary outcomes		
Blood analysis		
Serum 25(OH)D	*	*
Serum PTH	*	*
Serum calcium	*	*
Serum phosphor	*	*
Anthropometry and clinical assessment		
Height (cm), weight (kg)	* *	*
Body mass index (kg/m^2^)	*	*
Waist circumference (cm)	*	*
Body composition		
Fat mass (kg)	*	*
Lean mass (kg)	*	*
Body fat (%)	*	*
Visceral fat mass (kg)	*	*
Fat mass index (FMI)	*	*
Relative fat mass index (RFM)	*	*
Secondary outcomes		
Weight (kg), height (cm), head circumference (cm), of the infants	*	*
Infection frequency in infants		*
Frequency of doctor visits		*

### Sample size estimation

Sample size was calculated according to Roosta et al. [29] using two mean comparison formula. In this formula, considering α = 0.05, β = 0.2, and mean (standard deviation) of waist circumference changes after supplementation with vitamin D, which was equal to 1.91 cm (1.7) and 0.55 cm (1.04), respectively, in the intervention and control groups, sample size is estimated as 18 subjects. Since, there are three groups in this study, the calculated sample size was multiplied with $\sqrt{number\ of\ groups}$, and finally the desired sample size for this study was obtained as 90 subjects (30 subjects per group).

### Setting

This study will be conducted at a private hospital in Qazvin, Iran. Qazvin is located near the equator at a latitude of 50° 36′ N.

### Inclusion and exclusion criteria

Inclusion criteria are as follows:

1. Nursing women with overweight (BMI 25–29.9 kg/m^2^) or obesity (BMI 30–39.9 kg/m^2^)
2. Aged 20–49 years old
3. Delivered at term (gestational age of 37–42 weeks)
4. Normal birth weight (2500–3900 kg)
5. Declared exclusive nursing for the next 3 months

The exclusion criteria are:

1. Pregnancy
2. Having gastrointestinal disorders interfering with bowel function, severe hepatic, renal (dialysis), inflammatory, cancer, diabetes, hypertension, epilepsy, and thyroid diseases, or taking any medication
3. Regular intake of vitamin D supplements or multivitamins (once or more per week)
4. History of smoking or alcohol consumption once or more per week in the past month
5. Adhering to a specific diet during the past 3 months

### Randomization

This study is a double-blind, randomized, placebo-controlled, parallel trial. Subjects will be randomly assigned to three treatment groups using computer-generated codes. Randomization will be conducted by an assistant using permuted block randomization method and stratified randomization will be used to match the subjects based on age (20–34 and 35–49 years old) and BMI (25–29.9 and 30–39.9 kg/m^2^). Subjects will be randomly allocated into three groups of 2000 IU vitamin D/d (VD1), 4000 IU vitamin D/d (VD2), and placebo (P), and will be followed up for 12 weeks.

### Patient and public involvement statement

Patients and/or the public are not involved in the design, recruitment, and execution of the study. The main results will be disseminated to the subjects by email or telephone at the end of the study.

### Intervention

Vitamin D3 (as cholecalciferol) and placebo (as lactose) supplements are in the form of nano micro capsules and will be provided by the Nano Hayat Darou Industrial Co., Tehran. The capsules are identical in size, color, and shape. The present study will involve nursing mothers during autumn and winter. Ninety mothers who have just delivered will be invited to the study. Subjects will be randomly assigned to three groups to receive 2000 IU vitamin D/d, 4000 IU vitamin D/d, or placebo for 12 weeks. The length of intervention is selected based on similar clinical trials [30] and considering subject adherence.

Subjects will be required to consume one capsule with lunch and dinner. The intervention allocation will be blinded for both investigators and subjects. The double-dummy method will be used to double-blind the study. This means that the VD1 group will receive one capsule of vitamin D and one capsule of placebo per day. The VD2 group will receive two vitamin D capsules per day. Intervention will begin about 3 days after delivery and continue to 12 weeks later.

### Adherence and compliance

To assess the compliance of the subjects, they will be called every week. Subjects will receive supplements at the first visit and will be asked to bring all remaining ones for their last visit. Returned supplements will be counted to calculate the level of compliance and adherence to the intervention.

### Anthropometry and body composition

Body weight and height will be measured with subjects wearing minimal clothing and with bare feet. Weight will be measured to the nearest 0.1 kg using a calibrated digital weighing scale and height will be measured to the nearest 0.5 cm using a fixed stature meter (model number 26 SM). Waist circumference will be measured using a non-stretchable measuring tape from the point midway between the iliac crest and costal margin (lower rib) in the standing position. BMI will be calculated as weight in kilograms divided by the square of the height in meters. Bioelectrical impedance analysis (BIA) will be done using In Body model 270 as a body composition analyzer (In Body Co., Ltd, Seoul, Korea) to assess fat mass (kg), lean mass (kg), body fat (%), FMI as fat mass (kilogram) divided by the square of height (meters), and RFM calculated as:

$$ 76-\left(20\ \mathrm{height}\times \mathrm{waist}\ \mathrm{circumference}\ \mathrm{in}\ \mathrm{meters}\right) $$

BIA measurements are undertaken at least 6 h after breakfast. The subjects’ BIA measurements will be done while wearing light clothing and with bare feet and they will be asked to empty their bladders. The frequency used in this device is set as 20 and 100 kHz at each of the five body segments (right arm, left arm, trunk, right leg, left leg), and test duration is equal to 15 s.

### Dietary intake

To assess subjects’ dietary intake, two 24-h food recalls will be completed at baseline and the end of the study. One of the 24-h recalls will be filled for one of the working days of the week and another in the weekend. Pictures of food commonly consumed in Iran, together with a set of common household measurement tools (glass, cup, soup bowls, plates, teaspoon, and tablespoon), will be provided to assist subjects in estimating the portion sizes of the food.

The food items will be analyzed for their energy and nutrient content using the Nutritionist IV software version 4.1 (First Databank Division, the Hearst Corporation, and San Bruno, CA, USA), modified for Iranian foods. An Iranian food composition table will be used as an alternative for foods like Iranian bread (four items), cheese (two items), Kashk (whey), fruits (two items), sweets (nine items), and industrial fruit juice (one item) not included in the United States Department of Agriculture (USDA) food composition table [31].

### Sunlight exposure

A sunlight exposure questionnaire will be used to assess sunlight exposure. This questionnaire includes three questions covering the history of sunlight exposure, such as duration of outdoor activity in minutes per day, as well as sunlight avoidance history, such as use of an umbrella and sun block lotion. Subjects are also asked about their clothing style, such as wearing long sleeves and hijab or veil. The validity and reliability of the questionnaire have been reported in a previous study in Iran [32].

### Physical activity levels

The short form of the International Physical Activity Questionnaire (IPAQ) will be applied to assess the physical activity level of participants (http://www.ipaq.ki.se 2017). IPAQ is a seven-point questionnaire measuring the level of activity. In this questionnaire, the first and second questions are related to the number of days and intensity of physical activity, the third and fourth questions are related to the number of days and the average physical activity, the fifth and sixth questions are related to the number of days and light physical activity, and finally, the seventh question is related to the amount of time spent sitting in the last 7 days. The reliability and validity assessment of IPAQ across 12 countries showed that it can be used in many settings and in different languages [33].

### Blood sampling

Venous blood samples will be taken by the registered staff nurses between 9 and 10 am. The blood samples will be allowed to clot for about 60 min and will be centrifuged for 10 min at 2000 G within 2 h. All the blood serum will be stored at − 25 °C for further analysis. Blood serum will be used for analyzing calcium, phosphorus, PTH, and 25(OH)D3 levels, at baseline and the end of the study. Calcium and phosphorus levels will be measured by colorimetric enzymatic test (Pars Azmoon) and enzymatic photometric UV test BILT1500, respectively. For hormonal testing such as PTH, which need more precise testing, the Electrochemiluminescence (ECL) procedure, by Cobas e411 for Hitachi/Roche company, Germany, will be used. 25(OH)D3 will be measured by enzyme-linked immune sorbent assay (ELISA; Monobind Inc., Lake Forest, CA, USA).

### Statistical analysis

Missing data will be imputed using the regression imputation method. Quantitative variables will be reported as mean (standard deviation) and qualitative variables will be reported as percentages. ANOVA and Chi-square tests will be used to compare quantitative and qualitative variables in the three groups. To evaluate the effect of vitamin D supplementation on visceral fat mass, visceral fat percentage, lean mass, body fat percentage, waist circumference, FMI, RFM, BMI, and biochemical factors such as serum 25(OH)D, PTH, calcium and phosphorus levels two-way Repeated Measures ANOVA (RMA) will be applied in SPSS software version 21 (SPSS Inc., Chicago, USA). RMA assesses whether the mean changes in the results from baseline to 12 weeks differ in the three groups. This statistical analysis measures time to group interaction term. There are no planned interim analyses. The 95% confidence level will be considered.

### Patient safety

Patients will be monitored weekly during the intervention and any occurrence of adverse events will be reported. Moreover, to examine possible hypervitaminosis D, serum concentrations of calcium at the beginning and end of the study will be measured.

## Discussion

A large body of growing evidence shows that dairy products, calcium, and vitamin D play a role in regulation of body fat mass [34]. In addition, vitamin D may increase lean body mass [35] and inhibit the development of adipocytes [36]. These effects of vitamin D may be mediated by 1,25-(OH)D3 or through suppression of PTH [35]. However, there are no clinical trials on the effects of vitamin D supplementation on body composition among nursing women with overweight or obesity. Moreover, these kinds of multifactor studies are heterogeneous with regard to doses and types of vitamin D, length of follow-up, outcome ascertainment methods, prevalence of vitamin D deficiency, and other characteristics.

The main purpose of this study is to evaluate the role of vitamin D supplements in body composition among nursing women with overweight or obesity. We hope to provide guidance for the postpartum period, especially for mothers who are at risk of vitamin D deficiency, such as nursing women with overweight or obesity, to improve their vitamin D status by presenting the best dose of vitamin D supplement. This study also will provide more information on the effect of vitamin D on body composition. According to the high prevalence of vitamin D deficiency in pregnant and nursing women, these results may be used to convince governmental organizations providing free vitamin D supplements for these high-risk groups, to improve serum vitamin D levels, in addition to reduce the complications of vitamin D deficiency in nursing women.

### Strengths and limitations of the study

- The strengths of our protocol study include assessing the different doses of vitamin D supplementation to treat vitamin D deficiency in nursing women with BMI of 25 kg/m^2^ and higher.
- Also, this study is the first study evaluating the effects of different amounts of vitamin D supplementation on anthropometric status and body composition in nursing women with BMI of 25 kg/m^2^ and higher.
- Body composition measurements are not assessed by gold standard techniques, which can be considered as a limitation of this study.
- Possible poor cooperation of some patients is another limitation of this study.
- The calculated sample size is another limitation of this study. Due to financial constraints, the number of subjects is limited to 90. As such, a 10% dropout is considered the maximum.

### Trial status

The date of protocol registration was 04/11/2018, and the registration number is IRCT20140413017254N6. Recruitment will begin on 05/28/2018, and the approximate date when recruitment will be completed is 08/20/2018.

## Additional file

Additional file 1: The SPIRIT 2013 Checklist. (DOC 138 kb)

## Data Availability

Not applicable.

## Abbreviations

25(OH)D: 25-Hydroxy-vitamin D
ANOVA: One-way analysis of variance
BFP: Body fat mass percentage
BIA: Body impedance analyses
BMI: Body mass index
ECL: Electrochemiluminescence
ELISA: Enzyme-linked immunosorbent assay
FMI: Fat mass index
IOM: Institute of Medicine
IPAQ: International physical activity questionnaire
PTH: Parathyroid hormone
RFM: Relative fat mass
RMA: Repeated measures ANOVA
